# Egg Yolk Protein Water Extracts Modulate the Immune Response in BALB/c Mice with Immune Dysfunction Caused by Forced Swimming

**DOI:** 10.3390/foods11010121

**Published:** 2022-01-04

**Authors:** Mijoo Choi, Jae-Hoon Lee, Yun-Jung Lee, Hyun-Dong Paik, Eunju Park

**Affiliations:** 1 Department of Food and Nutrition, Kyungnam University, Changwon 51767, Korea; mijoo@kyungnam.ac.kr (M.C.); hjlee@kyungnam.ac.kr (Y.-J.L.); 2 Research Group of Food Processing, Korea Food Research Institute, Wanju 55365, Korea; leejaehoon@kfri.re.kr; 3 Department of Food Science and Biotechnology of Animal Resources, Konkuk University, Seoul 05029, Korea; hdpaik@konkuk.ac.kr

**Keywords:** immunomodulation, immunoassay, protein, egg yolk protein

## Abstract

The objective of this study was to determine the immunomodulatory effects of egg yolk protein–water extract (EYW) on splenocyte proliferation, cytokine secretion, immunoglobulin production, and NK cell cytotoxic activity in BALB/c mice. The forced swimming test (FST) was used to provide a model for suppressing immune regulation. The proliferation of B cells in the EYW supplementation group was significantly increased from the level to which it was reduced by the FST (from 40.9% to 81.8%, *p* < 0.05). EYW supplementation affected cytokine secretion of splenocytes. Levels of interleukin (IL)-2 and IL-10—as Th1 and Th2 cytokines, respectively—were decreased after the FST. However, EYW supplementation showed that secretion levels of these cytokines were significantly increased to pre-FST levels (*p* < 0.05). The production of immunoglobulins (IgA and IgG) was increased abnormally after the FST, whereas EYW supplementation significantly decreased it to pre-FST levels (*p* < 0.05). EYW supplementation also improved NK cell cytotoxic activity against YAC-1 tumor cells compared to the PC group (*p* < 0.05). These data suggest that EYW has potential as an immunomodulatory agent in the food and/or pharmaceutical industries.

## 1. Introduction

Immune regulation plays an important role in the immune system for disease prevention and treatment [1]. Impairment of immunity is expressed by marked susceptibility to pathogenic organisms. As a result, there is an increased risk of diseases such as allergy, malignancy, and autoimmune diseases [2]. Therefore, active substances with immunomodulatory and immunostimulatory activities have attracted the attention of many researchers [1,3,4]. Several pharmaceuticals, including dexamethasone, vincristine, and levamisole, can be used to control immune responses [5]. However, when using these pharmaceuticals in long-term therapy, there are unintended side effects, such as nausea, vomiting, high blood pressure, and stroke [6,7]. Daily intake of nutritional and functional foods is recommended in order to achieve healthy immune regulation. Many related studies have been conducted.

The forced swimming test (FST) is an animal behavioral test model that can measure antidepressant activity [8], and is used to test fatigue and endurance [9]; it can also be used to confirm immunomodulatory activity, because levels of factors involved in immune responses are changed after the FST [10]. Therefore, many studies on substances with immunomodulatory activities have used the FST to confirm that these substances can affect immune responses [11,12]. In order to confirm the immunomodulatory activity of a substance, its effects on splenocyte proliferation and inflammatory cytokine production should be determined.

Egg yolk contains many valuable components, including lipids, proteins, carbohydrates, and minerals; among them, lecithin is widely used in various industries. After separating egg yolk and lecithin, large amounts of byproducts are produced [13]. Because lecithin is obtained via ethanol extraction, these byproducts are mostly composed of proteins such as phosvitin, livetin, riboflavin-binding proteins, etc. [14]. These bioactive proteins possess various functional activities, such as antioxidant [15], anticancer [16], anti-inflammatory [17], and immunostimulatory activities [18]. However, these proteins are denatured and defatted during ethanol extraction procedures; thus, their value and functionality are limited [19]. To use these byproducts, various solvents (e.g., water, ethanol, acetone) can be used to extract bioactive compounds from them; among them, egg yolk–water extract (EYW) shows the strongest antioxidant activity (unpublished data). The aim of this study was to evaluate the effects of egg yolk–water extract (EYW) on proliferation, Th1/Th2 cell cytokine regulation, immunoglobulin production, and natural killer (NK) cell cytotoxic activity in primary splenocytes obtained from BALB/c mice with induced immune dysfunction caused by forced swimming.

## 2. Materials and Methods

### 2.1. Materials and Preparation of Egg Yolk Protein Extract

Roswell Park Memorial Institute (RPMI) 1640 medium and fetal bovine serum (FBS) were purchased from HyClone Laboratories, Inc. (Logan, MI, USA). Penicillin–streptomycin was purchased from Gibco-BRL (Grand Island, NY, USA). Lipopolysaccharide (LPS) and concanavalin A (ConA) were purchased from Sigma-Aldrich Co. (St. Louis, MO, USA). Murine ELISA kits for cytokine analysis were purchased from Bio Biosciences Co. (San Diego, CA, USA), while kits for immunoglobulin analysis were purchased from Immunology Consultants Laboratory Inc., (Portland, OR, USA). All other chemicals and reagents were purchased from Sigma-Aldrich Co.

The degreased egg yolk protein powder used in this study was obtained from Join Co. (Yongin, Korea). Egg yolk protein powder was extracted with water (EYW). Briefly, the EYW was extracted using an autoclave (DW-AC-131, Dong Won Scientific Co., Seoul, Korea) at 121 °C and 1.5× atmospheric pressure for 15 min by adding 100 mL of distilled water to 5 g of egg yolk protein powder. The solution was centrifuged at 1000× *g* for 10 min, after which the supernatant was freeze-dried for 3 days and stored at −20 °C until use.

### 2.2. Determination of Total Phenolic Content (TPC) and Total Flavonoid Content (TFC)

The TPC of EYW was determined using the Folin–Ciocalteu assay method [20]. Briefly, 400 μL of EYW was mixed with 400 μL of Folin–Ciocalteu reagent and kept for 5 min at room temperature. Then, 10% Na_2_CO_2_ was added to the mixture and kept for 1 h at room temperature. The absorbance was measured at 690 nm using a microplate reader (GEN5, BioTek Instruments Inc., Winooski, VT, USA), and the results were expressed as milligrams of gallic acid equivalents (GAE)/100 g of EYW.

The TFC of EYW was determined according to the method described by Moreno et al. [21]. Briefly, 100 μL of EYW was mixed with 1 mL of diethylene glycol and kept for 5 min at room temperature. Then, 1 M NaOH was added to the mixture and maintained at 37 °C for 30 min. The absorbance was measured at 420 nm, and the results were expressed as milligrams of quercetin equivalents (QE)/100 g of EYW.

### 2.3. Animals and Treatments (Experimental Animals)

**Figure 1:**
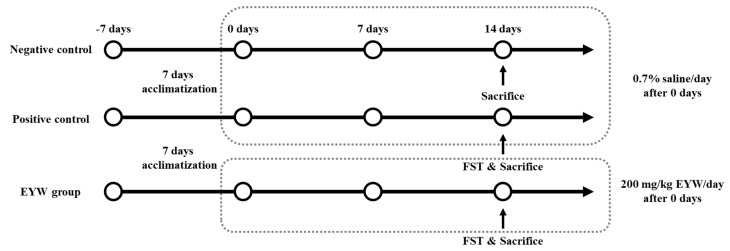
Animal experimental protocol.

The experimental protocol was approved by the Institutional Animal Care of Kyungnam University (KUIAC-18-04). Four-week-old male BALB/c mice were purchased from Koatech (Pyeongtaek, Korea). Mice were housed 3–4 per cage with a 12-h light/dark cycle at 23 ± 2 °C and 50% relative humidity. The basic feed was nutritionally complete (AIN-93G). Food and water were provided ad libitum. The animal experimental protocol is shown in Figure 1. Mice were acclimatized for a week before the experiment. A total of 24 mice were randomly divided into 3 groups (8 animals per group): negative control (NC, −FST), positive control (PC, +FST), and EYW (+FST). The animals in the EYW group were orally administered with 200 mg/kg body weight of EYW daily for 2 weeks. Meanwhile, the negative and positive control groups received 0.7% saline at the same volume via oral administration.

### 2.4. Forced Swimming Test (FST)

The forced swimming test (FST) is an experiment to confirm antidepressant or immunomodulatory effects, and measures the time that the experimental animals remain in a floating state in the water during the forced swimming [9]. The FST was conducted by placing animals in individual glass cylinders containing water at 25 °C, so experimental animals could not support themselves by touching the bottom with their feet. The average swimming time was calculated by checking the time of swimming by dropping out the experimental animals. The animals were removed from the cylinders, dried with paper towels, and then returned to their home cages. The forced swimming test was conducted once per week (2, 8 days), and swimming was performed before euthanasia for an average of 16 min.

### 2.5. Isolation of Primary Splenocytes from BALB/c Mice

Splenocytes were isolated via the method of Mishell and Shigi [22]. Obtained spleens were placed in RPMI 1640 medium. Single-cell suspensions were prepared by pressing the tissue through a 40 μm cell strainer (Falcon^®^, Corning Inc., Corning, NY, USA). Cells were centrifuged at 450× *g* and 4 °C for 5 min, and red blood cell lysing buffer (Sigma-Aldrich Co., St. Louis, MO, USA) was added to the pellets to obtain splenocytes. Splenocytes were maintained in RPMI 1640 medium supplemented with 10% FBS and 1% penicillin–streptomycin.

### 2.6. T-Cell and B-Cell Proliferation Assay

For determining the effects of EYW on the proliferation of splenocytes, a cell proliferation (WST; water-soluble tetrazolium) assay was used [23]. The splenocytes were plated in a 96-well plate (at 1 × 10^6^ cells/well), and then 5 μg/mL of LPS (a B-cell mitogen) or ConA (a T-cell mitogen) was added to each well. The plates were incubated at 37 °C in a humidified incubator containing 5% CO_2_ for 48 h. After incubation, 10 μL of EZ-Cytox (Enhanced Cell Viability Assay Kit, DoGenBio Co., Ltd., Seoul, Korea) solution was added and incubated for an additional 4 h. Absorbance was measured at 450 nm using a microplate reader. The cell proliferation was calculated as a percentage normalized to 100% of the splenocytes isolated from negative control group.

### 2.7. Th1- and Th2-Type Cytokine Production Assay

The Th1 (IL-2, TNF-α, IFN-γ)- and Th2 (IL-4, IL-6, IL-10)-type cytokines were determined using murine ELISA kits (Bio Biosciences Co.). Splenocytes were plated in 96-well plates (at 1 × 10^6^ cells/well), and LPS or ConA was added to each well at a 5 μg/mL concentration. The plates were incubated at 37 °C in a humidified incubator containing 5% CO_2_ for 24 h (for IL-2, IL-4, IL-6, IL-10, and TNF-α) or 72 h (for IFN-γ). Then, the secreted cytokine concentration of each well was quantitated by measuring absorbance at 450 nm.

### 2.8. Analysis of Immunoglobulins (IgA, IgE, and IgG) in Serum

Mice were fasted for 12 h and whole blood was collected from the retro-orbital plexus before euthanasia. After 20 min at room temperature, sera were obtained from whole blood using centrifugation at 2500× *g* and 4 °C for 30 min (Combi-514R, Hanil Scientific Inc., Seoul, Korea). The serum samples were used for the analysis of immunoglobulin (IgA, IgE, and IgG) production.

Immunoglobulins were measured using IgA, IgE, and IgG murine ELISA kits (Immunology Consultants Laboratory Inc.) in serum. Absorbance was measured at 570 nm using a microplate reader. Sera were diluted 1/4000-fold (IgA), 1/50-fold (IgE), and 1/50,000-fold (IgG), according to the kit protocol.

### 2.9. NK Cell Cytotoxic Activity

NK cell activity was measured using the CytoTox 96 Non-Radioactive Cytotoxicity Assay kit (Promega Corporation, Madison, WI, USA). NK cells, used as effector cells, were obtained from splenocytes, while YAC-1 cells, used as target cells, were obtained from the American Type Culture Collection (ATCC; Manassas, VA, USA). YAC-1 cells were cultured in RPMI 1640 medium supplemented with 2% FBS and 1% penicillin–streptomycin at 37 °C in a humidified incubator containing 5% CO_2_.

NK cells were added at 1 × 10^5^ cells/well, and YAC-1 cells at 1 × 10^4^ cells/well, at a 1:1 ratio. After incubation for 4 h at 37 °C, and centrifugation at 250× *g* for 4 min, the supernatant was used to measure lactate dehydrogenase (LDH) at an absorbance of 490 nm using a microplate reader. NK cell activity was confirmed by calculating the cytotoxicity of YAC-1 cells.

### 2.10. Statistical Analysis

All results are presented as the mean and standard error of three replicates. Differences between mean values of multiple groups were compared by one-way analysis of variance (ANOVA), followed by Duncan’s multiple range test. All calculations were performed using SPSS for Windows, version 20.0 (SPSS Inc., Chicago, IL, USA).

## 3. Results and Discussion

### 3.1. Preparation of EYW

In this study, egg yolk–water extract (EYW) was prepared using an autoclave, which are often used in many studies to extract functional substances from raw materials [24,25]. This autoclave extraction method has the advantage of being faster and more efficient than the conventional hot water extraction method [25].

The protein content of the EYW extracted using the autoclave was 28.9 ± 0.2 mg/g, the TPC content was 79.9 ± 0.9 GAE mg/100 g, and the TFC content was 5.8 ± 0.0 QE mg/100 g (Table 1).

### 3.2. Effect of EYW on Primary Splenocyte Proliferation

It is well known that murine primary splenocytes are mainly composed of B cells and T cells (41.5% and 47.1%, respectively) [26]. These primary splenocytes are used in many studies related to immune function, because they reflect the systemic immune response in vivo.

**Figure 2:**
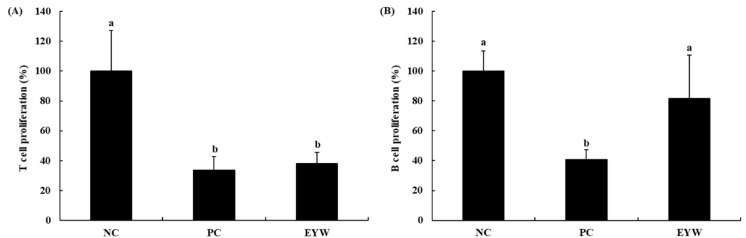
Effects of EYW intake on T/B-cell proliferation in splenocytes of BALB/c mice: (**A**) treated with concanavalin A (5 μg/mL); (**B**) treated with LPS (5 μg/mL). Data are presented as the mean ± SE of triplicate measurements. Various corresponding letters indicate significant differences at *p* < 0.05 according to Duncan’s multiple range test. NC: negative control (−FST); PC: positive control (+FST); EYW: egg yolk protein–water extract, 200 mg/kg (+FST).

The effect of EYW on the proliferation of primary splenocytes was determined based on a WST cell viability assay. LPS and ConA as mitogens for B cells and T cells, respectively, were used to treat splenocytes for 48 h; the cell viability of splenocytes was then measured (Figure 2). After the FST, the immune systems of mice become abnormal [11,12]. The PC group showed lower levels of proliferation of B cells and T cells (40.9% and 33.9%, respectively) than those observed in the NC group (100%). EYW supplementation significantly increased the proliferation of B cells (81.8%, *p* < 0.05) compared to the PC group, although it did not appear to restore the proliferation of T cells (38.2%). These results indicate that the administration of EYW can help to increase the immune response of mice.

The proliferation of splenocytes is well known as a crucial indicator of immune amplification. Increased proliferation of splenocytes is important for immunosuppressed mice, because splenocytes act as activators and regulators of immune systems [27]. Several studies have reported that the proliferation of splenocytes can be affected by immune-enhancing materials such as probiotics [28], proteins [29], and polysaccharides [3]. Yu et al. [5] reported that feeding peptides produced from fish skin in a 200 or 400 mg/kg diet for 3 weeks can increase the proliferation of splenocytes, thus confirming the immune-enhancing activity of such peptides. Similarly, low-molecular-weight peptides extracted from chum salmon have been found to be able to increase the proliferation of splenocytes in an immunosuppressed mouse model [30]. In addition, *Weissella cibaria* JW15 probiotics can increase cell proliferation and restore spleen weight and cell numbers in an immunosuppressed condition induced by cyclophosphamide [28]. Previous studies such as the aforementioned have demonstrated that splenocytes with increased activation and proliferation have a great effect on the production of cytokines, which play a major role in the immune system. Therefore, the effect on cytokine production caused by the increase in B-cell proliferation in the EYW supplement group was confirmed.

### 3.3. Effect of EYW on Cytokine Secretion by Primary Splenocytes from BALB/c Mice

The secretion of cytokines by splenocytes is an important process in immune responses. Cytokines secreted by splenocytes can be classified as Th1- and Th2-type cytokines. Th1 cytokines induce a pro-inflammatory response, whereas Th2 cytokines exert anti-inflammatory functions by inhibiting Th1 cytokine production [31]. Undoubtedly, a balance in the secretion of Th1/Th2 cytokines is very important in the immune system. Many studies on immune modulators have investigated their effects on cytokine secretion [32,33,34].

**Figure 3:**
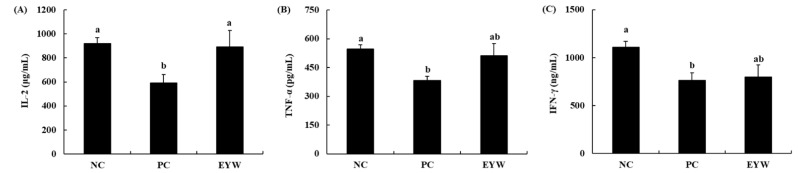
Effects of EYW intake on (**A**,**C**) ConA and (**B**) LPS processing Th1-type cytokines from splenocytes in BALB/c mice. Data are presented as the mean ± SE of triplicate measurements. Various corresponding letters indicate significant differences at *p* < 0.05 according to Duncan’s multiple range test. NC: negative control (−FST); PC: positive control (+FST); EYW: egg yolk protein–water extract, 200 mg/kg (+FST).

**Figure 4:**
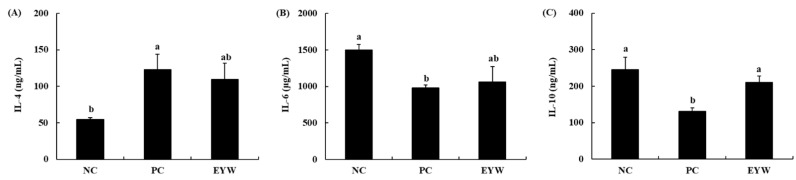
Effects of EYW intake on LPS processing Th2-type cytokines from splenocytes in BALB/c mice. Data are presented as the mean ± SE of triplicate measurements. Various corresponding letters indicate significant differences at *p* < 0.05 according to Duncan’s multiple range test. NC: negative control (−FST); PC: positive control (+FST); EYW: egg yolk protein–water extract, 200 mg/kg (+FST).

The effect of EYW on the secretion of cytokines by primary splenocytes was determined by ELISA. As shown in Figure 3, the secretion of Th1-type cytokines such as IL-2, TNF-α, and IFN-γ was significantly decreased in the PC group (591.6 μg/mL, 382.6 pg/mL, and 764.2 ng/mL, respectively) compared to the NC group (920 μg/mL, 547.7 pg/mL, and 1108.2 ng/mL, respectively, all *p* < 0.05). This meant that the forced swimming test induced immune suppression in mice. However, the EYW supplementation group showed a significant (*p* < 0.05) increase in IL-2 secretion compared to the PC group; the level of IL-2 secretion was 891.1 μg/mL, meaning that the immune suppression induced by the forced swimming test could be prevented by EYW supplementation. The secretion of TNF-α and IFN-γ in the EYW supplementation group also showed an increasing trend; however, this increase was not statistically significant compared to the PC group. Effects of EYW supplementation on the secretion of Th2-type cytokines are shown in Figure 4. The secretion of IL-4 was significantly increased after the forced swimming test (NC group: 54.6 ng/mL; PC group: 123 ng/mL), whereas secretion levels of IL-6 and IL-10 were significantly decreased in the PC group (980.6 μg/mL and 130.5 ng/mL, respectively) compared to the NC group (1498.2 μg/mL and 245.1 ng/mL, respectively) (both *p* < 0.05). However, the EYW supplementation group showed a significant increase in IL-10 secretion compared to the PC group (*p* < 0.05). For the secretion of other cytokines, there were no statistically significant differences between the EYW supplementation group and the PC group.

Cytokines play an important role in the immune system; they are known to regulate the communication between cells and participate in inflammatory reactions and cell proliferation [35]. Th1 cytokines secreted from Th1 cells can generally protect against intracellular infections caused by bacteria, viruses, and parasites by activating NK cells and macrophages [36], whereas Th2 cytokines secreted from Th2 cells can generally promote B-cell-mediated humoral immunity against extracellular pathogens [26]. IL-2 is a pro-inflammatory cytokine involved in the regulation of many immune cells, such as proliferation of T cells, B cells, macrophages, and NK cells [37]. IL-10 is an anti-inflammatory cytokine that can inhibit cytokine synthesis and reduce the release of reactive oxygen, thus reducing the ability of mononuclear phagocytes to present antigens [32].

Our results revealed that EYW supplementation could simultaneously promote the production of the Th1 cytokine IL-2 and the Th2 cytokine IL-10. This means that EYW could enhance immune responses by regulating the balance in the secretion of Th1/Th2 cytokines. Other researchers have found that some potential food components can simultaneously stimulate the production of Th1 and Th2 cytokines [4,34]. Han et al. [4] reported that polysaccharides obtained from barley leaves (*Hordeum vulgare* L.) showed immunomodulatory effects in immunosuppressed mice. These polysaccharides can markedly increase the secretion of both Th1 cytokines (IL-2 and IFN-γ) and Th2 cytokines (IL-10 and IL-4). Park and Lee [28] also reported that probiotics (*Weissella cibaria* JW15) can induce the production of inflammatory cytokines, which can be explained by increased proliferation of splenocytes. In our study, although FST-induced immune system disruption occurred, we also found that EYW supplementation increased B-cell proliferation and induced an increase in inflammatory cytokines.

### 3.4. Effect of EYW on Immunoglobulin Production

Serum levels of immunoglobulins IgA, IgE, and IgG were tested using ELISA kits. Immunoglobulin is secreted by plasma cells, and can neutralize foreign bacteria and viruses [33]; it plays a protector role by interacting with specific receptors and immune mediators [38].

As shown in Table 2, production levels of IgA and IgG in sera of the PC group (42.4 and 1459.8 μg/mL, respectively) were significantly increased compared to those in the NC group (31.9 and 583.6 μg/mL, respectively, *p* < 0.05). However, their levels in the EYW supplementation group (34.7 and 660.5 μg/mL, respectively) were significantly lower than those in the PC group (*p* < 0.05), but similar to those in the NC group. This means that EYW supplementation can help maintain the immune function impaired by the FST test. The production of IgE was not affected by the FST test or EYW supplementation.

IgA is the most abundant immunoglobulin in the body; it plays an important role in immune protection—especially in mucosal immunity—and can trigger effector functions to destroy invading microorganisms and mammalian cells [38]. IgG is the most abundant antibody isotype in the serum; it plays an important role in humoral immune responses, and can prevent the interaction between pathogens and host cells by binding to surface proteins of the pathogens [39]. Maintaining immune homeostasis is important for health; however, the homeostasis of immunoglobulin production was destroyed by the FST test, but adequately maintained by supplementation of EYW.

### 3.5. Effects of EYW on the Activity of Murine NK Cells

It is well known that NK cells can suppress tumor growth and metastasis effectively [40]. NK cells are present in peripheral blood monocyte cells (PBMCs) at 5–15%, and in splenocytes at 3–4% [41]. In the innate immune system, NK cells play a role as major cellular effectors to abrogate a variety of cells, including pathogenic bacteria, viruses, and tumor cells [42].

**Figure 5:**
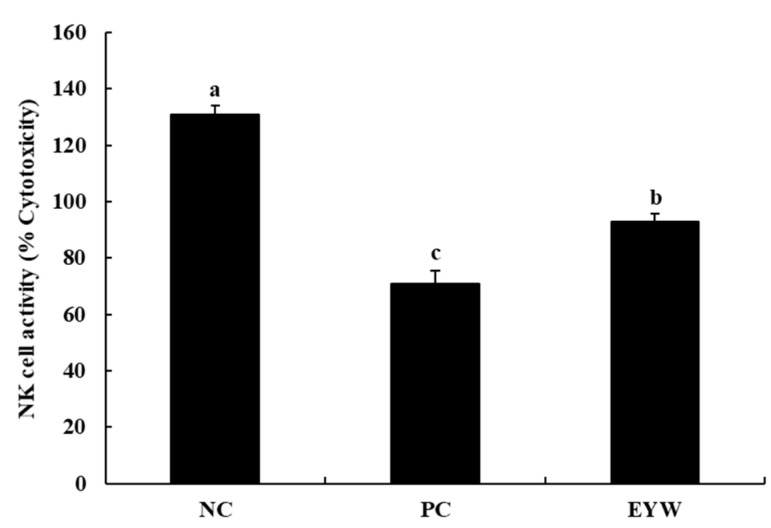
Effects of EYW intake on NK cell activity against YAC-1 from peritoneal macrophages in BALB/c mice. Data are presented as the mean ± SE of triplicate measurements. Various corresponding letters indicate significant differences at *p* < 0.05 according to Duncan’s multiple range test. NC: negative control (−FST); PC: positive control (+FST); EYW: egg yolk protein–water extract, 200 mg/kg (+FST).

We investigated the effects of EYW on the NK cell activity of splenocytes by evaluating levels of lactate dehydrogenase (LDH), which is known to be released from damaged cells; the results are shown in Figure 5. Compared to the NC group (130.9%), the PC group showed lower NK cell activity (70.79%), indicating that the FST test induced a decrease in the NK cell activity of splenocytes. However, EYW supplementation significantly increased NK cell activity (92.9%) compared to the PC group (*p* < 0.05), although NK cell activity in the EYW group was not the same as that in the NC group. Similar to our study, Park, Oh, Kim, and Choi [43] reported that *Corchorus olitorius* leaf extract showed immunomodulatory activity by increasing NK cell activity in immunosuppressed mice. Shin, Hwang, Yoon, Kim, and Shin [44] also reported that ginseng leaf polysaccharides can increase NK cell cytotoxic activity against YAC-1 cells, and can help to maintain the immune system. It is well known that Th1 cytokines are involved in NK cell activation [37]. Several studies have reported that IL-2 can increase the cytotoxic activity of NK cells [45]. In the present study, the EYW supplementation group showed a protective effect against the reduction of IL-2 cytokine production by the FST test. For this reason, it is considered that the cytotoxic activity of NK cells in the EYW supplementation group increased compared to the PC group.

## 4. Conclusions

The present study provides strong evidence that egg yolk protein–water extract (EYW) can be used as an immunomodulatory agent in the food and/or pharmaceutical industries. The results of the animal experiments in this study showed that EYW supplementation could help to restore the immune system after deterioration caused by the FST test. EYW supplementation increased B-cell proliferation and increased the secretion of IL-2 and IL-10, which had been decreased by FST. After EYW supplementation, production levels of IgA and IgG were restored to their levels prior to the FST test. Finally, reduced NK cell activity of splenocytes was increased by EWY supplementation.

## Figures and Tables

**Table 1:** Protein, total phenolic, and total flavonoid contents of EYW.

	Protein Contents (Protein mg/g)	Total Phenolic Contents (TPC, GAE mg/100 g)	Total Flavonoid Contents (TFC, QE mg/100 g)
EYW	28.9 ± 0.2	79.9 ± 0.9	5.8 ± 0.0

**Table 2:** Effects of EYW intake on immunoglobulin production in the sera of BALB/c mice.

	NC	PC	EYW
IgA (μg/mL)	31.9 ± 0.6 ^a^	42.4 ± 0.4 ^b^	34.7 ± 0.2 ^a^
IgE (ng/mL)	81.3 ± 4.6 ^ns^	96.7 ± 5.4	89.0 ± 6.8
IgG (μg/mL)	583.6 ± 32.3 ^a^	1459.8 ± 317.7 ^b^	660.5 ± 26.6 ^a^

Values are the mean ± SE. For all values of each sample, different superscripts within a row mean significant differences at *p* < 0.05 according to Duncan’s multiple range test. NC: negative control (−FST); PC: positive control (+FST); EYW: egg yolk protein–water extract, 200 mg/kg (+FST). ^ns^: not significantly different between groups.

## Data Availability

Not applicable.
